# Items of General Interest

**Published:** 1916-02

**Authors:** 


### Items of General Interest.

The elegant country home of Dr. W. F. Odom, of Kurten, was recently destroyed by fire.

---

After a long struggle the Sisters of Mercy have succeeded in the establishment of their hospital at Laredo.

---

Negotiations are under way for a $500,000 hospital in connection with the Texas Christian University at Fort Worth.

We have information that La Grange is soon to have a surgeons' hospital.

---

A company is being organized to build a large sanitarium at Kenedy to utilize the hot water from the hot well. The water has been proven to have curative power.

---

The Waco Baptists have purchased a site for a $100,000 sanitarium they are to build. Rev. J. M. Dawson, former pastor of the Hillsboro Church, is head of the movement.

---

An association composed of the Baptist Churches of Waco have purchased a block of ground at the corner of Twenty-fifth and Bernard Streets, and will erect a Baptist sanitarium.

---

It is held that misbranding and false statements as to the curative effects of medicines in interstate commerce can be punished as lottery tickets. Numerous actions against drug concerns are expected to follow.

---

The last option on property covering ten acres was secured in the heart of Hot Springs, Ark., on which a $10,000,000 sanitarium is to be erected. This sanitarium will be one-third larger than anything of its kind in the world.

---

The Fort Worth board of health will be asked to take up with the city commission the matter of having the water mains of the city given a thorough cleaning, as the result of action taken by the Doctors' Luncheon Club at the Westbrook.

---

Outlined plans of the proposed Oak Cliff Hospital soon will be presented to the Dallas County Medical Association for approval by a special committee from the Oak Cliff Medical and Hospital Association. It is said every physician in Oak Cliff approves the plans and has given personal endorsement to the proposition.

---

The successful transplanting of the nerves of young pigs into the human body has been reported to the Moscow Society of Surgeons by Dr. D. A. Gruzdeff. Three operations, all of which have good results, were described by Dr. Gruzdeff. In two cases he had to deal with a leg that had been paralyzed by a wound; in the third case with an arm that had been similarly paralyzed.

A nose which in line and symmetry compares favorably with the natural variety has been manufactured from a soupbone by surgeons at Samaritan Hospital at Philadelphia for a 12-year-old girl. An incision was made in the frontal bone of the skull and the soupbone nose securely fixed in position. Then the flesh of the face was stretched so that it covered the soupbone. Nostrils were pierced in the flesh, and the child was able to breathe freely through the new nose.

---

The State Board is very desirous of securing the number of cases of leprosy and their location in the State of Texas. An effort is being made to establish a National Leprosarium, and there are no statistics on this disease that are available. While this disease is by law notifiable, the physicians have overlooked the reports. Physicians are requested by the State Board to secure a complete report from their county and mail it at once to the Health Department at Austin.

---

The Secretary of the Association of Military Surgeons of the United States and editor of the *Military Surgeon* announces that the headquarters of the Association and of the *Military Surgeon* have been transferred from Chicago to Washington, D. C., and requests that hereafter all communications, periodicals for exchange, books for review, etc., be addressed to the newly elected Secretary-Editor, Lieutenant Colonel Edward L. Munson, M. C., U. S. Army, or to the Association.

---

A ward in which patients sleep on inflated rubber mattresses half submerged in tubs filled with warm water is the latest feature of the famous open-air military hospital at Cambridge, England. The water which is kept flowing through the tub is maintained at a temperature of 100°.

One patient whose thigh had been ripped away by a shell has been in a tub continuously for six weeks. Before he was placed in the tub he said he feared he was going insane from the pain, but during his sojourn in the water he had not felt anything worse than the usual discomfort from long confinement in bed.

---

Dr. P. W. Covington of the International Health Commission, with headquarters in New York City, was in Austin recently and conferred with Dr. W. B. Collins, State Health Officer, relative to inaugurating in Texas a co-operative plan to better health conditions. The commission co-operates with the State, county and city health boards in an effort to improve health conditions throughout the country.

Dr. Covington expects to visit the counties of Middle and Eastern Texas and take up the matter with the various commissioners courts as well as with the county boards of health.

---

Drs. F. G. Benedict and F. E. Talbot of the nutrition laboratory of the Carnegie Institution of Boston announce to the National Academy of Science in its proceedings for December the results of a study on the physiology of the new-born infant.

They find that the new-born infant is not supplied with a superfluous amount of carbohydrate (starches and sugar) to burn during the earlier period of insufficient flow of milk, and the predominance of the consumption of body-fat suggests the possible necessity of supplemental feeding of carbohydrates.

Profound disturbances of the temperature are found to accompany the bath of the infant following its birth, and there are great influctions in the heat values for the first day. This makes it worth while considering whether it might not be well to defer the bath for a day to permit the infant's heat-regulating mechanism, which appears to be deficient at birth, to have a long period of adjustment to the new conditions of external life.

